# Transactions of the Medical Society of the County of Kings

**Published:** 1865-10

**Authors:** 


BUFFALO

### Medical and Surgical Journal.

VOL. V. OCTOBER, 1865. No. 3.

ART. I—*Transactions of the Medical Society of the County of Kings.*

### REGULAR MEETING, APRIL, 1865.

### *Otitis, Perforating of the Mastoid Process with a Trephine.*

REPORTED BY DR. J. C. HUTCHISON.

Henry Steele, a seaman, 23 years of age, entered the Brooklyn City Hospital May 31st, 1859. Four weeks previously he had an abscess behind the ear which was opened, discharged pus freely and then healed. This was followed by a purulent discharge from the ear, for which he entered the hospital. He had no pain after coming into the house until June 13th, when he was attacked with intense ear-ache and tenderness on pressure over the mastoid process. The pain was excruciating, and was not at all relieved by the abundant use of anodynes internally, anodyne fomentations, counter irritants, etc. On the 15th I found him still suffering intensely, and suspecting that the pain arose either from an abscess in the mastoid cells, an accumulation of pus in the cavity of the tympanum, or from inflammatory swelling of the mucous membrane which lines the cavity of the tympanum and mastoid cells, I at once determined to perforate the latter with a trephine.

After exposing the outer surface of the mastoid process by a crucial incision, the crown of the trephine was applied to the bone, inclining it forward and upward in proportion as it penetrated until the auditory cells were reached. No pus was found, nor was the process diseased. A probe introduced into the wound passed without obstruction from the mastoid cells into the cavity of the tympanum. The wound was closed with silver sutures, and water dressing applied.

The operation gave entire relief at once; he ate and slept well, and indeed suffered no farther inconvenience, except a slight discharge from the ear. The wound healed kindly by granulation, and he left the hospital July 20th.

The prompt relief afforded in this case was owing no doubt to the removal of the tension caused by the swelling of the membrane which lines the unyielding bony walls of the tympanum and mastoid cells.

*Epithelial Cancer of the Glans Penis.*

Dr. Hutchison also presented a specimen of *Epithelial Cancer of the Glans Penis* with the following history: Charles Dozle, Ireland, age 60, presented himself for treatment in October, 1864, complaining of pain about the glans penis, scrotum and perineum. He had congenital phymosis, but an enlargment could be felt through the foreskin on the under part of the glans penis which was sensitive to the touch. His general health had suffered somewhat on account of the pain which was so severe at night as to prevent sleep. I advised slitting up the prepuce for the purpose of examining the tumor more satisfactorily. He would not consent to the operation and subsequently consulted Dr. Mott and one or two other eminent surgeons without being benefitted. He applied to me again early in March and consented to have any treatment adopted that I deemed proper. I exposed the glans by dividing the prepuce and found an elevated graunlar surface on the left side of the glans, which presented the appearance of epithelial cancer, bleeding readily when touched.

I advised removal of the organ, which was done with a scalpel, a short distance behind the glans. Four arteries were tied and the mucus membrane of the urethra was divided by the knife into four equal parts which were tacked to the edges of the cutaneous portion of the wound. The object of this procedure was to prevent contraction of the orifice of the canal which is so liable to follow the ordinary operation.

On the following day he said that he had slept better the previous night than for a number of months. With the exception of retention of urine after the third day, which necessitated the introduction of a catheter into the bladder several times, no untoward symptom followed the operation, and he now feels quite well.

The diseased portion has lost its color and is also diminished in size by the alcohol in which it has been preserved. The specimen was examined under the microscope by Dr. Spier, and was found to be epithelioma.

---

### REGULAR MEETING, MAY, 1865.

### *Report of a Case of Hydrophobia.*

BY R. C. STILES, M. D.

John McE., aged 23, a native of Baltimore, a currier by trade, well built, and of temperate habits, was brought to the hospital about noon on Friday, May 5th. From his own statement and that of his friends, the following history was obtained:—

He had been bitten by a dog on the Monday morning previous. The dog was morose, disposed to be let alone and snapped at those who disturbed him, but was not supposed to be mad. He was killed four days afterwards. The patient was bitten on the back of the hand; the wound was very slight. On Monday night he felt slight pain in the hand but slept well. The following morning the pain had extended to his arm and shoulder and he could not drink his tea—attempted to take it through a tobacco pipe, but could not swallow it. He ate however a crust of bread; but neither ate nor drank again till he entered the hospital. On Wednesday night he had pain in his chest with difficulty of breathing, was raving and furious so that it took several persons to keep him in bed. He was seen by a physician on Wednesday evening who pronounced the case one of hydrophobia. A physician from New York saw him on Thursday afternoon, and pronounced the case one of hydrophobia; ordered an injection.

*Symptoms while in the hospital.*—The *puls*[illegible]n admission was 160 per minute, became more frequent and after four hours was imperceptible.

The *blood* examined under the microscope presented an increased proportion of white corpuscles, about one of the white to twenty of the red.

The *lungs* on auscultation were found sound, but almost every inspiration was interrupted by a spasmodic closure of the glottis producing a sobbing respiration which continued till death.

The *skin* was covered with a cold sweat. The temperature in the axilla an hour after admission was 92° Fahr. The heat of the body rose with the application of warmth to the lower extremities, which were quite cold at first. The tongue was furred; he was constantly spitting a clear liquid ; on taking some toast soaked in wine and sugar he vomited and continued to vomit whatever he took till an hour before death. He used a gutta percha tube to draw some tea into his throat to quench his thirst, but he vomited it continually. He stated that he had passed his urine and fœces involuntarily since Tuesday. There was a slight involuntary discharge of urine while he was in the hospital. The most interesting phenomena, however, were those presented by the nervous system.

*Sensation* was affected in the right arm, (the one bitten,) which felt numb, and did not recognize painful impressions as readily as the other. There was no tenderness on percussion or pressure over the spine; no tenderness over the abdomen except in the loins, which I attributed to the soreness from previous convulsions. The spinal senses were very acute long after the pulse ceased to be felt. The eyes were red and protruding. He said he felt them protrude. The sound of running water distressed him, and caused a shiver to pass through his frame. Draughts of air or the lighting of flies on the face produced a similar effect, but he complained that he could not breathe under a musquetoe-net. There were constant twitchings of the muscles of the face, these moving the alo nasi, and the lips in particular. His right hand was paralyzed; when he moved in bed he moved the right arm by the aid of the left. All his movements were sudden, even turning in bed.

His *intellect* was clear, and he answered questions with remarkable precision till ten minutes before his death. He raved, however, for about an hour before death. He had the fearfulness of delirium tremens. When water was mentioned he repelled it with an intense expression of terror. He dreaded the approach of night for he said he then saw ugly sights. Long after the pulse had entirely failed he leaped from the bed and fell on the floor, excited by a light having been brought near him.

Death at 11 P. M., in an attack of vomiting—*Post-mortem* 11 hours after death. *Rigor mortis* extreme, particularly of the bitten arm.

*External appearance.*—Diffused purple discoloration of posterior surface of trunk and limbs; eyes staring; scar of bite almost imperceptible. *Brain* normal. *Spinal cord* and its meninges deeply congested—possibly from subsidence of the blood. *Heart* unusually rigid. *Lungs*—Liver and spleen normal. *Kidneys* somewhat congested. *Bladder* empty. *Intestines* deeply congested. *Pharynx* and upper portion of æsophagus slightly congested. *Stomach* contracted. *Blood*, fluid.

This case was a type case of hydrophobia not to be confounded with delirium tremens, tetanus, or other nervous disorder; commencing within twenty-four hours after the bite of a rabid dog. Death occurred through failure of the heart's activity, and the symptoms pointed to the upper portion of the spinal cord as the seat of lesion of the central nervous system. No treatment whatever was employed except the applications of heat to the lower extremities.

